# Challenges to the operation of Brazilian LTCIs and changes in oversight

**DOI:** 10.1186/s12877-024-05129-4

**Published:** 2024-06-13

**Authors:** Letycia Parreira de Oliveira, Henrique Salmazo da Silva

**Affiliations:** Programa de Pós-Graduação Stricto Sensu em Gerontologia. Brasília, Universidade Católica de Brasília, Distrito Federal, Brasil

**Keywords:** Long-term care institution for older adults, Older adults, Care, Health care, Policy

## Abstract

**Background:**

Despite 18 years since health surveillance regulations were promulgated in Brazil to govern Long-Term Care Institutions for Older Adults (LTCIs), many institutions fail to comply with the Differentiated Regime for Public Procurement (Resolution No. 502/2021) due to structural and operational conditions. This study aimed to investigate Brazilian LTCI managers’ understanding of challenges that significantly impact institutional operation and gather suggestions for enhancing RDC No. 502/21.

**Methods:**

A cross-sectional, exploratory, and qualitative study was conducted, involving 90 managers or technical supervisors from Brazilian LTCIs. Data were collected using a self-administered Google Forms instrument and analyzed through Thematic Analysis based on the Organizing for Quality (OQ) framework.

**Results:**

The most impactful challenges for LTCIs were healthcare, financing, human resources, relationship with oversight bodies, and family members.

**Discussion:**

Proposed improvements for RDC No. 502/21 included enhanced professional training, infrastructure revision, increased financial support from the state, realistic oversight/regulations, and tailored monitoring approaches.

**Conclusion:**

LTCIs in Brazil face numerous challenges, and the suggested improvements aim to adapt regulations to institutional realities. However, considering the regulations’ variability and purposes, further investigation is warranted.

**Supplementary Information:**

The online version contains supplementary material available at 10.1186/s12877-024-05129-4.

## Background

Population aging is an undeniable fact and one of the greatest challenges of the 21st century. It is estimated that by 2050, 1/4 of the world’s population will be composed of individuals aged 60 or older, with 1/3 of this population presenting Non-Communicable Chronic Diseases (NCDs) and care demands [1]. In Brazil, according to a special report issued by the *Departamento Intersindical de Estatística e Estudos Socioeconômicos* [2], out of the total of 210 million Brazilians in the fourth quarter of 2021, 37.7 million were older adults, corresponding to 18.5% of the country’s population [2, 3]. Unlike some countries, Brazil has witnessed an accelerated process of population aging, becoming the sixth country in terms of the number of older adults. Thus, the aging of the Brazilian population urges the State, public managers, and civil society to plan strategic methods of care and attention to older [4].

In Latin America, historically, the role of the caregiver has been exclusively composed of family members, but this care may no longer be sufficient to meet all the biopsychosocial needs of aging [5]. Therefore, the State plays a fundamental role in the development and implementation of effective public policies and formal care outside the family context, such as those provided by Long-Term Care Institutions for Older adults (LTCIs) [6]. LTCIs are defined by the *Agência Brasileira de Vigilância Sanitária* (ANVISA, Brazilian Health Surveillance Agency) as “governmental and non-governmental residential institutions, intended for the collective domicile of individuals aged 60 or older, with or without family support, in conditions of freedom, dignity, and citizenship” [7].

As stated by ANVISA [7], institutions are responsible for providing a cozy and homelike environment, accompanied by a technical team, individual care, and healthcare services. Although institutionalizing older adults is not yet part of Brazilian culture, this care modality is one of the most prevalent among the population of older adults, especially among the oldest old [4]. It is also typified in the policy of high-complexity social protection, ensuring the rights, autonomy, welcoming, family interaction, and humanization in the care of older adults [8].

After numerous cases of mistreatment in institutions, in order to establish a minimum operating standard for LTCIs in Brazil, *Resolução da Diretoria Colegiada* (RDC, Resolution of the Collegiate Board) No. 283 of 2005 was created, currently repealed by RDC No. 502 of May 27, 2021 [9]. This RDC established the guarantee of the rights of this population according to the legislation, the obligation to prevent and reduce health risks for institutionalized older adults, as well as the need to improve the provision of public and private services in LTCIs, covering various aspects of these, from minimum operating standards to monitoring the services provided, including detailed requirements such as the characteristics of the external and internal flooring, minimum distance between beds in rooms, availability of technical norms, routines for food storage procedures, waste disposal, among others. However, many institutions have failed to meet the established criteria, facing challenges in implementing the outlined regulations [10]. In practice, Brazilian institutions struggle with unstable financial support and the precariousness of care offerings from the state. Philanthropic institutions make up the majority in the Brazilian scenario regarding LTCIs, with approximately 65% of them. Some institutions cater to individuals who are not older adults, and it is estimated that there are over 7,000 institutions throughout the national territory, with variations in organizational profiles and operating conditions [11].

Furthermore, in Brazil, the lack of integrated long-term care policies between social assistance and healthcare hinders the potential functioning of LTCIs, putting them at a disadvantage in the face of the high degree of complex care demanded by the population of older adults [12, 13]. Although LTCIs do not have an exclusive focus, purpose, or designation as healthcare services, they provide healthcare assistance based on the functional limitations and degree of dependency of the older [14]. Therefore, it is evident that changing the culture of long-term care involves the provision of policies that make institutions more welcoming, humane, and with greater sources of financial investment in order to offer higher-quality care.

In this process, knowledge of the practices within institutions is essential for establishing recognition and quality systems in the care of older adults, as monitoring through indicators has not yet been fully implemented and should be considered a priority. Efforts by the International Association of Gerontology and Geriatrics, together with the WHO, have been made to describe the major challenges faced by institutions and outline possible guidelines to overcome them. According to the Organizing for Quality (OQ) framework, the analysis of various factors such as structure, policy, culture, education, emotions, physical and technological factors, health, financing, human resources, relationship with oversight bodies, and relationship with the family are important aspects to consider in the provision of care [15].

Based on this theoretical-conceptual model, the objective of this study was to investigate the understanding of managers or technical supervisors in Brazilian LTCIs regarding the challenges that most impact their functioning and to gather suggestions and proposals for the improvement of RDC No. 502/21. Although the data is specific to Brazil, in developing countries where long-term care is not yet sufficiently established as public policy, the importance of these institutions is even more significant, and they are mostly philanthropic. However, the insights presented can support the development of actions and reflections for long-term care in countries with similar contexts where care still lacks investments. Thus, the data resonates with the reality of other developing countries. In this discussion, understanding the opinions of technical supervisors and managers who work in these services can contribute to the improvement of policies, programs, and services in Brazil, as well as aligning with the technical-scientific development of interventions and management processes in human aging.

## Method

This is a cross-sectional, exploratory, and qualitative study. Qualitative research involves obtaining descriptive data through direct contact between the researcher and the studied situation, trying to understand phenomena from the perspective of the subjects or participants in the study situation [16]. It also demonstrates the specific ability to systematically explore the contents to be developed, while gaining insights into phenomena, experiences, and human behaviors, thus facilitating a comprehensive understanding of the participants, which is fundamental in the field of gerontology [17].

### Sample

We investigated a sample of managers or technical supervisors from public, private, and philanthropic LTCIs, and the final sample size was estimated considering the number of institutions listed in the *Sistema Único de Assistência Social* (SUAS, System of Unified Social Assistance) Census of 2015/2016 [18, 19]. The formula used was n = N.Z².p.(1-p)/Z².p.(1-p). + e².(N-1), where “n” is the calculated sample size, “N” is the population size, “Z” is the standard normal variable associated with the confidence level, “p” is the true probability of the event (P=(1-P) = 0.5, assuming maximum variation), and “e” is the sampling error.

Based on the 1,280 institutions surveyed by the SUAS Census and using a sampling error of 5% and a confidence level of 95%, the aim was to investigate a sample of 296 technical supervisors and/or managers of each institution. However, after sending invitations to the National Front for Strengthening LTCIs, as well as to Municipal and State Councils for older adults, the final sample consisted of 90 managers and/or technical supervisors of LTCIs. It is worth noting that Brazil lacks an information system that consolidates data on LTCIs, and neither does it deeply understand the reality of managing these institutions. Thus, we relied on estimates of institutions surveyed in the Census of the System of Unified Social Assistance, but it remains an approximation. Therefore, the studied sample is not representative as it did not reach the desired sample size (*n* = 296). However, there are currently no studies that have investigated the theme explored by the present study, focusing on RDC 502/2021. The research methodology involved inviting a representative sample of managers and/or technical managers to participate in the study, in which they were spokespersons for the LTCIs, meaning that for each participant there is an institution to be researched [20].

The National Front for Strengthening LTCIs was created in April 2020, during the COVID-19 pandemic in Brazil, as a matter of urgency, with the aim of assisting institutions in promoting strategic planning, activities, and research, in addition to strengthening ILPIs to create long-term public care policies [21]. To this end, in collaboration with the National Front for Strengthening LTCIs, as well as the Municipal and State Councils for Older Adults, as well as the LTCIs, the final sample was made up of 90 managers and/or technical managers from each institution [19].

The participants were both men and women with internet access and varying degrees of educational levels. The institutions to which they belonged were required to meet the following criteria: (a) have been in the same physical location for at least two years; (b) have a Technical Supervisor in charge of the work; and (c) agree to participate in the study by signing the Informed Consent Form (ICF).

### Instruments

In this study, a self-administered instrument was used, consisting of nine variables and 35 objective and a discursive question, and made available through an electronic form on the Google Forms platform for managers and technical supervisors. This is an excerpt from the general research, in which we used two questions from the instrument that was also used in other studies. The instrument was developed based on the inspection guidelines of ANVISA and, above all, the domains of RDC No. 502 of 2021. It was reviewed by scholars and experts in long-term care to encompass as many items as possible that are part of the quality and management of services. After receiving contributions, the instrument was finalized and formatted. Therefore, the questions were constructed considering the opinions of experts and the theoretical framework on Brazilian LTCIs, such as Camarano’s study (2010), which presents the most comprehensive census of institutions to date, and subsequently more updated studies on the challenges and profiles of LTCIs. A systemic approach to management was considered, involving everything from human resources to the core activities of the services. The research instrument script includes questions relating to structure, policies, culture, education, emotions, physical and technological factors, health, finances, human resources, relationship with supervisory bodies and relationship with the family (APPENDIX A). The areas that cover the OQ aim to provide a comprehensive and structured context of the challenges within the institution, allowing knowledge of the processes and practices in assisting elderly people [15]. For the topic, two questions were asked about the challenges that impact the functioning of LTCIs and proposals for improving RDC502/21 according to managers and technical managers of LTCIs. To broaden the discussion, managers and technical supervisors had the opportunity to record, through an open-ended question, what other challenges most impact the functioning of LTCIs.

### Procedure

The sample was recruited using the snowball technique, which is characterized as a non-probabilistic and convenience sampling technique, where individuals selected to be studied invite new participants from their network of friends and acquaintances. The use of this sampling technique aimed to obtain the maximum number of participants and overcome the barrier of distrust surrounding research involving institutions. Snowball sampling is a commonly employed sampling method in qualitative research. Regarding the sample size of 90, it represents the maximum number of responses obtained after numerous invitations and promotions during the six-month period of the year 2021 (second semester). It is worth noting that during this period, Brazil was experiencing the COVID-19 pandemic, and many institutions faced significant operational challenges. Despite the limitations of this method, it was necessary due to the unfeasibility of face-to-face instruments, due to the COVID-19 pandemic. Furthermore, there is participation bias. That is, many institutions consider that studies on organizations or inspections are investigative inquiries [20].

Invitations were made between May and December 2021 through phone calls, emails, WhatsApp, and YouTube. Upon receiving the invitation, participants who agreed to participate were provided with the link to the self-administered instrument along with the informed consent form and the instrument’s questions. The research instruments were applied in July/December 2021, a time when vaccination measures were ongoing during the COVID-19 pandemic.

### Data analysis

The data from the open-ended questions were categorized according to the thematic analysis proposed by Cecília and Carlos [22]. This type of analysis allows for establishing a bundle of relationships on a specific subject, represented by phrases, words, or summaries. It involves discovering the core meanings that compose a communication, whose presence or frequency signifies something related to the study’s objective. Thematic analysis is the process of identifying patterns or themes within qualitative data, making it a very flexible method, being a considerable advantage given the diversity of work in learning and teaching. This is much more than simply summarizing data; a good thematic analysis interprets and makes sense of it. A common pitfall is to use the main interview questions as the themes [23]. Typically, this reflects the fact that data have been summarized and organized, rather than analyzed. In this way, out of an instrument with 14 questions, only two open-ended questions were used, which addressed the challenges impacting the operation of LTCIs and proposals for improving RDC502/21 according to LTCI managers and technical staff. The challenges referenced by the participants were organized into categories that grouped the main challenges of LTCIs according to the managers and technical supervisors of Brazilian LTCIs, as well as suggestions and proposals for improving RDC No. 502/21.

### Ethical aspects

This study was submitted to and approved by the Ethics Committee of Catholic University of Brasília, CAE protocol No. 39648820.0.0000.0029.

## Results

In this study, 90 institutions were investigated, with over half situated in the Southeastern region (51.1%), followed by the Midwestern (26.7%), Northeastern (14.4%), and Southern regions (7.8%). In terms of institutional nature, the majority identified as private non-profit (34.4%), followed by philanthropic institutions with an SUAS agreement (26.7%). Additionally, 25.6% represented for-profit private institutions, with only 8.9% identified as public institutions. In terms of the profile of the investigated TSs (Technical Supervisors) and LTCI managers, most participants were female (84.4%), with a postgraduate degree (43.3%), and holding a position related to LTCI management, with their length of service ranging from one to six years (54.4%).

### Testimonies from technical supervisors and managers about other challenges that impact the functioning of LTCIs

According to the thematic analysis by [22], the participants’ responses were grouped into five categories: health, financing, human resources, relationship with oversight bodies, and family.

#### Health care services offered by LTCIs

Regarding the Health category, the participants stated:

*“The institution pays for exams and consultations that are not covered by the Unified Health System.” LTCI,1*.

*“What hinders us the most is the issue of the health of older adults, as the Unified Health System has a lot of delays in specialist appointments and exams.” LTCI,6*.

#### Financing of LTCIs

In the Financing category, the following statements are highlighted:

*“Our biggest difficulty is financial.” LTCI, 40*.

*“Older adults should be fully funded by the state since many families rely on the 30% contribution. And the cost of caring for an older person is high.” LTCI, 58*.

#### Human resources of LTCIs

Regarding the Human Resources category, some managers reported that:

*“There are many employees who take a job solely to ‘earn’ unemployment benefits” LTCI, 3*.

*“There are many companies looking for good professionals, but many lack motivation because they prefer to simply obtain unemployment benefits.” LTCI, 38*.

#### Relationship with oversight bodies

About the relationship with oversight bodies, the following statements were selected:

*“The extreme rigidity of some oversight bodies in enforcing RDC No. 283.” LTCI, 22*.

*“Disinformation from health surveillance staff regarding the assessment of older adults’ quality of life.” LTCI, 27*.

#### Family relationships

In the context of family presence in the care environment, the participants stated:

*“Family caregiver partnership, presence, and acceptance of other family members.” LTCI, 8*.

*“Family conflicts.” LTCI, 25*.

#### Proposals for improving RDC No. 502/21 according to LTCI managers and technical supervisors

The participants’ responses were grouped into four categories: Improvement and training of professionals working in LTCIs, revision of infrastructure for routine activities, need for greater financial support from the state, and more realistic proposals for oversight/regulations and increased monitoring within the reality of each LTCI. These categories align with the education, physical and technological factors, policy, and culture issues proposed by the OQ framework.

#### Improvement and training of professionals working in LTCIs

Regarding the human resources domain, the professionals specifically proposed:

*“Include the obligation of having nursing professionals in the staff.” LTCI, 45*.

*“Professional with a higher education degree and post-graduate qualification in Health and Gerontology for Older Adults, Regulation of LTCIs (preventing clandestine operations).” LTCI,21*.

#### Infrastructure review for routine activities

About the infrastructure, the following proposal was made:

*“Review the issue of bedrooms, not limiting the quantity, but rather the size of the space in relation to the number of older adults who reside in the institution.” LTCI, 5*.

#### More realistic inspection/standard proposals and greater inspection within the reality of each LTCIs

Regarding the characteristics of the institution and documentation, the following was mentioned in the reports:

*“RDC No. 283 should differentiate between private, public, and philanthropic LTCIs due to the differences and specificities existing in each legal registration of each LTCI.”LTCI,11*.

*“Equalize and balance the evaluation criteria for all institutions, regardless of the number of residents.”LTCI,51*.

*“Each institution is unique and always seeks improvements; however, the RDC does not always align with the reality we experience and ends up causing frustration for not being able to comply with the regulations.”LTCI,3*.

*“Propose realistic solutions rather than idealistic ones.”LTCI,6*.

Related to this, proposals for improving the offerings are also considered, such as:

*“Humanization in the analysis of criteria, prioritizing the care and comfort of older adults, as well as preserving their human dignity in the provision of care.”LTCI,44*.

*“Aligning legislation in accordance with the reality of existing LTCIs, prioritizing the well-being and good care of older adults instead of focusing solely on structural and human resources, and ensuring that oversight bodies maintain criteria and requirements that are feasible for the institutions.”LTCI,54*.

*“Projects that enable government support for LTCIs,”LTCI,88*.

*“Lack of financial contribution from the Municipal and Federal Governments.”LTCI,21*.

## Discussion

For each category (health, financing, human resources, relationship with oversight bodies, and family), connections were established with the themes proposed by the OQ framework, which can contribute to reflections on improving care in Brazilian institutions.

### Health care services offered by LTCIs

In the Health category, according to the testimonies, older adults who resided in LTCIs did not receive all the necessary health support. According to Creutzberg et al. [12], there is mutual unfamiliarity between LTCIs and the *Sistema Único de Saúde* (SUS, Brazilian Unified Health System), requiring better structural integration [24]. In Brazil, although the healthcare system is universal, there is still no specific policy for providing care to institutionalized older adults [25].

Considering that the LTCIs population is frail and has different healthcare demands, the lack of healthcare assistance raises concerns about the quality of care. In recent years, this discussion has been revived with the COVID-19 pandemic, including the planning of a continued care policy. However, the integration between SUS and LTCIs requires multifaceted efforts and multidimensional interventions [25], which necessitate the presence of healthcare professionals, who are often absent from these institutions.

### Financing of LTCIs

In the Financing category, the statements regarding financial scarcity are highlighted. According to the Brazilian Statute of Older adults [25], institutions can only keep up to 70% of their financial assistance provided by the state, which means that, if the institution receives 100% of this benefit, it will be against what is legally allowed e according narrative of LTCI 58. However, the relationships surrounding the income of older adults should be subject to further analysis. According to the participant’s account, the family is the receiver of the 30% that would be allocated to the older adults for their needs, which competes with the institution’s financial difficulties. This highlights the need for a broader debate on the financing of long-term care and how to ensure the rights of institutionalized older adults [26].

According to Poltronieri et al. [27], violations in LTCIs can be attributed to the absence of public policies focused on Brazilian long-term care, which fail to guarantee the necessary financing, support, and protection for these institutions. This includes negligence in care within the institution or among its residents. As well as conditions preceding institutionalization, such as the scarcity of gerontological services in the community, and situations of violation within the socio-familial context [27]. Consequently, LTCIs are the last and often the only resort for older adults facing situations of abandonment and mistreatment due to the scarcity of services and the absence of state support. However, upon entering LTCIs, violence may persist due to the lack of structural conditions for providing care, inadequate training of professionals, and insufficient monitoring of violence cases in these institutions.

### Human resources of LTCIs

Regarding the challenges identified in the Human Resources category, some managers report that many professionals who work in LTCIs are only there temporarily. This is because they are simply waiting for the minimum required period to become eligible for state-provided unemployment benefits, which entail receiving monthly payments for a predetermined period without the need for employment.

It is also noteworthy that the LTCIs are not charity institutions; they are businesses, and like any business, they have employer-employee relationships. Hiring requires a selection process that may involve instrument’s questions and resume analysis to identify the most suitable professional for the required position [28].

The *Consolidação das Leis de Trabalhistas* (CLT, Consolidation of Labor Laws) regime consolidates and regulates various aspects of labor rights and obligations, including employment contracts, working conditions, wages, benefits, labor disputes, and social security provisions. Thus, all employees CLT hired by the institution result in expenses such as salary, vacation pay, thirteenth salary, and social charges, such as severance fund and social security contributions [29]. Additionally, it is worth noting that there are still prejudiced beliefs and stigmas associated with LTCIs. There is a prejudice in linking these institutions to places of imprisonment, abandonment, poverty, or where older adults stay because they do not have a family available to care for them [30]. However, social stigmatization is being overcome with the provision of good public and private services.

It is also important to highlight that the work carried out by professionals in LTCIs is permeated by a daily and exhausting workload. That can affect not only their physical, mental, and emotional health but also the quality of care provided to institutionalized older adults [31]. Moreover, low salaries and the accumulation of tasks make it difficult for professionals to find pleasure in their work [32]. In this context, it is important not only to evaluate the caregivers’ health-related quality of life to promote actions and programs that can support them [33] but also to provide continuous training for these healthcare professionals. The real need for training is included in the guidelines of the Brazilian Policy on the Health of Older Adults, as it promotes the education of healthcare professionals as a strategy for the health care of older adults [25].

### Relationship with oversight bodies

The relationship with oversight bodies is also a challenge faced by LTCIs. In Brazil, the relationship between institutions and Health Surveillance seems to adopt a punitive approach. Although this dynamic is perceived as complementary, the imposition of penalties is generally viewed negatively. However, every inspection carried out in LTCIs is based on regulations, aiming to evaluate the quality of care and assistance provided, as well as to identify any potential health risks to the residents considering their vulnerability. Countries like the United States are starting to discuss regulatory rules again. Over 90% of institutions in the US that are enrolled in Medicare and Medicaid must comply with over 170 quality and health standards established by their legislations. States can contribute with additional norms.

Concerns regarding the quality of care have persisted for many years in an attempt to gather efforts to regulate specificities in the care of institutionalized older adults [26, 34]. In addition, the US Department of Health and Human Services proposed that nursing homes participating in Medicaid or Medicare must provide information about the quality of care and general conditions to service centers and the state. This initiative strengthens transparency in long-term care for older adults, allowing oversight bodies to improve the services provided through educational information to the institutions [28].

Initially, in Brazilian institutions, rigidity reflects the difficulty of complying with what is established in RDC No. 502/21 [35]. There is evidence of partial compliance with the current regulations, both in terms of physical-structural and organizational aspects. It sometimes exposes older adults to inadequate conditions, posing risks to their health. According to the guidelines set forth by the RDC, older adults are to be followed-up according to their degree of dependence, and a comprehensive care plan should be developed for each individual. However, many institutions do not have established internal norms and routines, further hindering the implementation and application of the RDC [36].

Therefore, it is necessary for institutions and oversight bodies to promote adjustments and educational interventions to comply with the current legislation. It also generates discussions about the feasibility, implementation, and conditions of institutional care. Given the other challenges faced by managers and the scope of the RDC, it is evident that training those who are in charge, as well as the team of employees, is necessary to comply with the legal provisions [9].

Actions of continuing education have shown to be effective in improving care for older adults, as they lead to improvements in the procedures performed by the staff of LTCIs. It is important to emphasize the value of Permanent Health Education (PHE) for those responsible for enforcing the RDC, as opposed to punitive actions. It is believed that oversight bodies should interact with managers and technical supervisors of LTCIs in order to address the gaps highlighted in our findings [37].

### Family relationships

In the context of family presence in the care environment, it is important to highlight that adequate family involvement in the lives of older adults in LTCIs is not always a reality. According to Creutzberg et al. [24], it is expected that there is a mutual care relationship between the resident’s family and the LTCI. However, due to a lack of inclusion strategies and communication failures, the family does not always participate in the daily actions of the institution, and the role of maintaining emotional ties is hindered [24]. The very admission of the older adults into the institution may indicate the existence of weakened or broken family bonds. It requires work on strengthening and redefining those bonds [38, 39]. In this sense, LTCIs, in the presence of specialized professionals, could foster and develop this by establishing a commitment and sense of partnership between them for the successful transition of the older adults to the institution. Therefore, it is necessary to develop strategies that promote a union between LTCIs and families, fostering more meaningful family connections and establishing more effective interactional systems [24].

According to thematic analysis by [22], the responses to the improvement proposals were categorized into four groups: improvement and training of professionals working in LTCIs, revision of infrastructure for routine activities, the need for greater financial support from the state, and more realistic proposals for inspection/norms and increased inspection within the reality of each LTCI.

### Improvement and training of professionals working in LTCIs

The reality of human resources in Brazilian LTCIs is characterized by a team with a wide range of qualifications, as in some institutions, the composition is linked to the financial condition of the family, caregiver, or the older adults’ own resources to hire such services [40].

The constant pursuit of improvement in long-term care extends to several countries, including Germany, which has one of the highest numbers of older adults who reside in institutions. Understanding the current situation of care provided by nursing homes is important for enhancing end-of-life care. Specific information about the reasons for the admission of older adults and knowledge of palliative care are identified as crucial areas that highlight the need for a larger number of staff in nursing homes and efficient training for the team [41].

Many institutions do not have an adequate quantity of Nurses, impacting the provision of safe and harm-free care to the older adults and affecting both the care provided and the health of the professionals. Additionally, in Brazil, institutional managers often lack training in the field of aging. This contrasts with countries such as the United States where specific training in Gerontology is required to be the manager of an LTCI. In this context, institutional management is advised to leverage technical-scientific expertise in structuring and implementing comprehensive care for older adults. This entails being grounded in management processes, negotiation skills, and prioritizing the needs of residents, the professional team, families, and other stakeholders involved [40, 42].

### Infrastructure review for routine activities

According to the reports, the infrastructure is not always in compliance with regulations. The inadequacy of the dormitories compromises the privacy of older adults, often disregarded or inappropriately incorporated into the environment. As a result, personal and territorial space is not recognized, which can negatively impact the perception of individuals and activities within the institution [43].

### More realistic inspection/standard proposals and greater inspection within the reality of each LTCIs

Regarding the characteristics of the institution and documentation, the need to understand the different realities of each LTCI is mentioned. It is important to highlight, regarding the type of institution, contradictory opinions regarding evaluation criteria. The first respondent argues for differentiation in evaluation based on the nature of the institution (public, private, or philanthropic). The second respondent supports the idea that institutional evaluation criteria should be similar.

In practice, according to the RDC, institutions are evaluated equally. In the discussion about possible differentiation, it is not clear which parameters and specificities should be considered. For example, if we already know that most institutions in Brazil are philanthropic and face financial difficulties, how can the *Superintendência de Vigilância Sanitária/ Agência Nacional de Vigilância Sanitária* (SUVIS/ANVISA, Superintendence of Health Surveillance/National Health Surveillance Agency) relax the rules if they are based on sanitary operating standards? If the institution falls under the category of a collective residence, are the criteria not sufficiently elucidated regardless of the nature?

According to a study by Fagundes et al. [44], some private institutions showed unsatisfactory food and habitability, public institutions had a high prevalence of unsatisfactory habitability. Philanthropic institutions had a prevalence of notifications for uncontrolled diseases [44]. Therefore, it is necessary to investigate the organizational and resource conditions associated with all types of institutions.

In the face of the many needs of older adults, relevant aspects such as ensuring and exercising their rights, their social life, and their personal well-being need to be discussed in order to create an ideal living scenario for older adults. Thus, to achieve excellent results in different areas, joint efforts by the State and the families are extremely necessary. The State needs to take care of aspects related to legislation, retirement plans, and public policies for older adults, while the family needs to provide care and support. This perspective aligns with what was envisioned by the Brazilian Federal Constitution of 1988, which assigns the responsibility of ensuring a dignified life to both the State and the Family [45].

The issue of financial support is also a frequently highlighted problem. In the Brazilian context, financing is scarce, making it urgent to expand the role of municipalities and states in allocating resources to institutions. The option for the government to offer financial assistance to LTCIs is due to the greater ease it finds in transferring income rather than providing services. It would be necessary for the government, through SUS, to establish mechanisms for greater control of the management of resources allocated to these institutions. While philanthropy alleviates community demands on one hand, on the other hand, it hinders the construction of an adequate public policy for care and the recognition of care as a social right [46].

Leadership and management play a crucial role in improving the quality of care and safety for older adults. LTCIs have the potential to provide solid professional services and constantly work towards improving patient and user quality and safety. Quality management through management systems brings challenges for managers and technical supervisors, such as planning, organizing, implementing, and improving services in LTCIs. Therefore, it is essential for managers and technical supervisors to understand the challenges and barriers to quality and safety in long-term care [47].

The limitations of this study include limited access to managers and technical supervisors from the Northern region of the country, a small sample size attributed to low participation in the research, and potential response bias stemming from the use of an electronic form. Despite these limitations, the presented results are the first on changes in the sanitary surveillance system of Brazilian institutions. Understanding the thoughts of professionals and technicians helps in shaping changes in the supply system and attention to institutionalized older adults. Furthermore, the research is particularly important when considering the difficulties that many institutions have experienced during the COVID-19 pandemic, which has claimed many lives among the institutionalized population of older adults in Brazil.

## Conclusions

The understanding of managers or technical supervisors in Brazilian LTCIs regarding the challenges that most impact the functioning of these institutions involved categories such as health, financing, human resources, relationship with oversight bodies, and family. Suggestions and proposals for improving RDC No. 502/21 included the improvement and training of professionals working in LTCIs, revision of the infrastructure for routine activities, the need for greater financial support from the government, more realistic proposals for regulation/norms, and increased oversight tailored to the reality of each LTCI.

The data align with the OQ framework. Understanding the international standard of care (OQ) as the goal to be achieved, different challenges experienced by managers and technical supervisors in these institutions in Brazil were identified. The investigation highlighted areas that could be changed from the perspective of managers and technical supervisors in collaboration with oversight bodies. It became evident that certain points hinder the application of sanitary regulations, although it was not possible to pinpoint which regulatory points need to be altered. The proposed changes suggest adaptations to the reality of the institutions, but given the variability and purpose of the regulations, this subject deserves further exploration. Therefore, it can be concluded that there are many challenges for LTCIs in Brazil. Addressing these challenges will require short, medium, and long-term actions, with a particular emphasis on the involvement of gerontologists and professionals committed to the quality of care for institutionalized older adults.

### Electronic supplementary material

Below is the link to the electronic supplementary material.

Supplementary Material 1

## Data Availability

The datasets used and/or analyzed during the current study are available from the corresponding author on reasonable request.

## Abbreviations

LTCIs Long: Term Care Institutions for older adults
NCDs Non: Communicable Chronic Diseases
ANVISA: Brazilian Health Surveillance Agency
RDC: Resolution of the Collegiate Board
SUAS: System of Unified Social Assistance
ICF: Informed Consent Form
OQ: Organizing for Quality
SUS: Brazilian Unified Health System
CLT: Consolidation of Labor Laws
PHE: Permanent Health Education
SUVIS: Superintendence of Health Surveillance
