# Supplementary material for: Challenges to the operation of Brazilian LTCIs and changes in oversight
Source: BMC Geriatr. 2024 Jun 13;24:515. doi: 10.1186/s12877-024-05129-4 (PMC11177510; doi:10.1186/s12877-024-05129-4)
Supplement: Supplementary file 1 — Supplementary Material 1 [file 12877_2024_5129_MOESM1_ESM.docx]

**APPENDIX A: Form - Manager and/or Technical Responsible - ADAPTED**

**CONTENT VALIDITY ASSESSMENT**

We invite you to evaluate the content of the instrument RDC 283/2005: the challenges in caring for the elderly, the understandings and changes in the guidelines that govern the functioning of Brazilian LTCFs.

The instrument aims to evaluate managers/technical managers and professionals who work with elderly people to verify the challenges and understandings in the care and management of LTCFs in Brazil. To this end, it was based on the regulations established by RDC 283 of ANVISA of 2005, as well as on issues focused on managing and facing institutional challenges.

The evaluation of the instrument involves 2 phases, detailed below:

1) general evaluation of the instrument;

2) evaluation of the domains;

3) evaluation of items.

1. Domain evaluation: We ask that you first evaluate the domains. Check that the domain structure and its content are correct, that the content contained in the domain is representative and that it is appropriate for respondents. Therefore, consider the concept of coverage as described below in your assessment: Comprehensiveness: check whether each domain or concept was adequately covered by the set of items. During this phase, you can suggest the inclusion or exclusion of items in the domains and give your opinion on whether the items really belong to the corresponding domain.

2. Assessment of items: In the second stage, we ask you to evaluate each item separately, considering the concepts of clarity and relevance/representativeness as described:

• Clarity: evaluate the wording of the items, that is, check whether they were written in such a way that the concept is understandable and adequately expresses what is expected to be measured;

• Relevance or representativeness: note whether the items really reflect the concepts involved, whether they are relevant and whether they are suitable for achieving the proposed objectives. Use the agreement scale to evaluate these criteria, marking an X in the corresponding field. Below each scale, we leave spaces so you can write suggestions to improve the item, suggest inclusion and/or elimination of items, or make comments. Furthermore, you can view the new instrument as an attachment.

• Coverage: check whether each domain was adequately covered by the set of items and whether all dimensions were included.

Use the yes or no options, or the scale from 1 to 4 to evaluate these criteria, marking an X in the corresponding field. Below each scale, we leave space for you to write suggestions or make comments.

I. Evaluate the title for clarity (check whether it adequately expresses what is expected to be measured).

TITLE: RDC 283/2005: challenges in caring for the elderly, understandings and changes in the guidelines that govern the functioning of Brazilian LTCFs

Is the title of the instrument clear and expresses the measure?

1 = not clear

2 = unclear

3 = quite clear

4 = very bright

II. Evaluate the format (lay-out) for clarity (check that the format is understandable) and suitability.

Is the format understandable?

1 = not clear

2 = unclear

3 = quite clear

4 = very bright

III. Evaluate the instructions for clarity (check that the wording is correct and adequately expresses what is expected to be measured).

Are the instrument instructions clear?

1 = not clear

2 = unclear

3 = very clear

4 = very light

IV. Step 1

[Transform the word questionnaire into PDF, and then each domain into a JPEG file. Paste the JPEG file into each Google Form question]

Does the above domain express the content? ( ) Yes No

Should items in Domain 1 remain in that domain? ( ) Yes No

Is Domain 1, in general, comprehensive?

1 = not comprehensive

2 = needs major revision to be comprehensive

3 = needs little revision to be comprehensive

4 = comprehensive

Comments

VII. Step 2 - Evaluate each Domain __ item for clarity (wording of the items, if they were written in such a way that the concept is understandable and adequately expresses what is expected to be measured) and representativeness (note whether the items really reflect the concepts involved, whether they are relevant and whether they are suitable for achieving the proposed objectives).

[Insert the jpeg file for each question]

Are the items in Domain 1 of the instrument clear and understandable? ( ) Yes ( ) No. Comments:

If yes, how much?

1 = not clear

2 = unclear

3 = very clear

4 = very bright

Comments:

Are the items in Domain 1 representative of the concepts explored, are they relevant?

( ) Yes ( ) No. Comments:

If yes, how much?

1 = not clear

2 = unclear

3 = very clear

4 = very bright

Comments:

Are the items in Domain 1 of the instrument clear and understandable? ( ) Yes ( ) No. Comments:

If yes, how much?

1 = not clear

2 = unclear

3 = very clear

4 = very bright

Comments:

Survey on the application, challenges and changes in RDC 283/2005, which governs the functioning of Brazilian LTCs.

**FORM – MANAGER AND TECHNICAL RESPONSIBLE**

| **IDENTIFICATION DATA** |
| --- |
| 1. Municipality: |
| 1. Region of Brazil: ( ) North ( ) Northeast ( ) Central-West ( ) Southwest ( ) South |
| 1. Status: |
| 1. Year of founding of the Institution: |
| 1. The nature of the institution is:   ( ) Public  ( ) Private for profit  ( ) Private non-profit  ( ) Philanthropic without an agreement with the Unified Social Assistance System  ( ) Philanthropic with an agreement in the Unified Social Assistance System  ( ) Other _________________________ |
| 1. Position or Function in the Institution: ( ) Technical Manager ( ) Manager |
| 1. How long have you been working at the Institution? ( ) less than 1 year ( )1 to 6 years ( ) 7 to 10 years. ( ) more than 10 years. |
| 1. Do you have another role at LTCs: ( ) Yes. Which one? _____________ ( ) At the |
| 1. Education: ( ) Elementary education ( ) Secondary education. Specify ______ |
| ( ) University education. Specify______ ( ) Postgraduate degree. Specify ______ |
| 1. How do residents enter the institution? |
| ( ) Via Public Ministry |
| ( ) CRAS/CREAS |
| ( ) Waiting list |
| ( ) Spontaneous demand |
| ( ) Others . Specify :____________________________________________________________________ |
| 1. Does the institution have non-admission criteria for residents? If so , which ones ?   ( ) LGBTQIA+ audience  ( ) Alzheimer's disease and other dementia syndromes  ( ) Psychiatric illnesses  ( ) Infectious diseases  ( ) Degree of Dependence I  ( ) Degree of Dependence II  ( ) Degree of Dependence III  ( ) No benefits/retirement or income  ( ) Others. Specify:_ ___________________ |

| **CLIENT CHARACTERISTICS** | |
| --- | --- |
| 1. How many residents does your institution currently have? | |
| men | Women |
| Total: | |
| 12.1 Do you have any transsexual or transvestite residents? If yes , please specify _________________ | |
| 1. What is the total number of residents that can be accommodated in LTCs? | |
|  | |
|  |  |
| Note: If all elderly people in the institution are classified in the same degree of dependence, mark an X in the corresponding box. | |
| 1-Grade I- Independent elderly people even if they use self-help equipment. |  |
| two- Grade II - Elderly people with dependence on three activities of daily living such as: eating, mobility, hygiene; without cognitive impairment or with controlled cognitive alteration. |  |
| 3- Grade III - Elderly people with dependence who require assistance in all self-care activities for daily life and/or cognitive impairment. |  |

|  | | |  |
| --- | --- | --- | --- |
| **GENERAL CONDITIONS AND OPERATIONAL PROCESSES OF THE INSTITUTION** | | |  |
|  | | |  |
| 14 Select YES or NO if your institution has: | SIM | NÃO | |
| Health Surveillance Permit |  |  | |
| Habitability Report |  |  | |
| Fire Department inspection |  |  | |
| Work plan with institutional activities and offers; |  |  | |
| Bylaws; |  |  | |
| Internal Regulations; |  |  | |
| Registration of the social entity; |  |  | |
| Updated record of each old person; |  |  | |
| Health Care Plan; |  |  | |
| Registration with the Municipal Council for the old adults or COMA |  |  | |
|  |  |  | |
| Others: | | |  |
| 1. What is the institution’s main source of financing? (Which guarantees more than 51% of LTCs expenses) | | |  |
| \| ( ) Convênio SUAS \| ( ) Retenção de até 70% do benefício ou aposentadoria do usuário \| \| --- \| --- \| \| ( ) Pagamento direto pelo usuário \| ( ) Retenção de 71 a 100% do benefício ou aposentadoria do usuário  ( ) Mantenedora \| \| ( ) Outros \| \| \| ( ) Não se aplica \| \| |  | |  |
|  |  | |  |
|  | | |  |
| 16 A instituição possui outras fontes secundárias de financiamento?   \| ( ) SUAS Agreement \| ( ) Retention of up to 70% of the user's benefit or retirement \| \| --- \| --- \| \| ( ) Direct payment by the user \| ( ) Retention of 71 to 100% of the user's benefit or retirement  ( ) Maintainer \| \| ( ) Outros \| \| \| ( ) Não se aplica \| \| | | |  |
| 1. Você considera que as fontes de financiamento são suficientes para cobrirem os gastos?   ( ) Totalmente ( ) Parcialmente ( ) Mais ou menos  ( ) Pouco ( ) Muito pouco | | |  |

| **HUMAN RESOURCES** | | | | |
| --- | --- | --- | --- | --- |
| 1. What is the composition of the professional team that works at LTCs ? | | | | |
| **Team** | **Amount** | **Comments** | | |
| Caregivers |  |  | | |
| Nursing Technicians |  |  | | |
| Cleaning Professionals |  |  | | |
| Kitchen/food professionals |  |  | | |
| Nurse |  |  | | |
| Physiotherapist |  |  | | |
| Social Worker |  |  | | |
| Psychologist​ |  |  | | |
| Other professionals, please specify: | | | | |
| 1. What is the main work regime of the professional team?   [Select one or more alternatives that best correspond to the reality of your institution] | | | | |
| ( ) CLT | ( ) MEI (Individual Micro Entrepreneur) | ( )Contract for the provision of independent services | | |
| ( ) Others | | | | |
| 20. With regard to professionals who provide direct and indirect care to residents, the institution: | | | YES | NO |
| Has 1 caregiver for every 20 elderly people Dependency Degree I, or fraction, with a workload of 8 hours/day | | |  |  |
| Has 1 caregiver for every 10 elderly people Level of Dependence II, or fraction thereof, per shift; | | |  |  |
| Has 1 caregiver for every 6 elderly people Level of Dependence III, or fraction, per shift | | |  |  |
| It has a Technical Manager working 20 hours per week. | | |  |  |
| There is one cleaning professional for every 100m2 ^of^ internal area or fraction thereof per shift daily. | | |  |  |
| It has one food professional for every 20 elderly people, guaranteeing coverage of two eight-hour shifts. | | |  |  |
| It has a professional laundry service for every 30 elderly people, or fraction thereof, daily. | | |  |  |
| The staff has a health professional duly registered with its professional council. | | |  |  |
| 20.1 Does your institution have or develop: | | | YES | NO |
| Volunteer Program | | |  |  |
| Permanent Education and Continuing Training Program on Gerontology | | |  |  |
| Case discussion meetings | | |  |  |
| Standard Operating Procedures Manuals, if so, which ones?  - Food  - Cleaning  - Nursing and resident care  - Others | | |  |  |
| 1. you consider that Human Resources are sufficient to offer assistance to residents?   ( ) Completely ( ) Partially ( ) More or less  ( ) Little ( ) Very little | | | | |

| **INFRASTRUCTURE** | | |
| --- | --- | --- |
| 1. Do you consider that the physical structure of the institution and the property are suitable for offering assistance to residents?   ( ) Completely ( ) Partially ( ) More or less  ( ) Little ( ) Very little | | |
| 1. As for Infrastructure, check if your institution offers it. | YES | NO |
| It has an architectural project for construction, renovation or adaptation of the physical structure in accordance with approval by the local health authority as well as the competent municipal body. |  |  |
| Provides physical facilities in livable, healthy, safe and accessible conditions |  |  |
| It has ramps to facilitate access and movement for residents. |  |  |
| Meets Brazilian technical standards regarding building water and sewage installations |  |  |
| Meets Brazilian technical standards regarding electrical energy |  |  |
| Meets Brazilian technical standards regarding fire protection and fighting, telephony and others |  |  |
| **Regarding access** | YES | NO |
| It has at least two external access doors, one of which is exclusively for service. |  |  |
| It has external and internal floors (including ramps and stairs) that are easy to clean and maintain, uniform, with or without joints and with an anti-slip mechanism. |  |  |
| It has Ramps and Stairs made in accordance with the specifications of NBR 9050/ABNT, observing the requirements for handrails and signage. |  |  |
| It has stairs at least 1.20m wide. |  |  |
| It has main internal circulations with a minimum width of 1.00 m and secondary circulations can have a minimum width of 0.80 m; counting on permanent wake light. |  |  |
| It has handrails in circulations with a width greater than or equal to 1.50 m on both sides; |  |  |
| It has a handrail on only one side with a circulation space of less than 1.50. |  |  |
| The doors have a free span with a minimum width of 1.10m, with simple locking without the use of locks or keys. |  |  |
| Windows and railings have sills of at least 1.00m. |  |  |
| The elevators follow the specifications of NBR 7192/ABNT and NBR 13.994. |  |  |

| 24 As for other environments, your institution has: | | |
| --- | --- | --- |
|  | Yes. How many? | No |
| Ecumenical and/or meditation space. |  |  |
| Administrative/meeting room |  |  |
| Dining room with a minimum area of 1m ^2^ per user |  |  |
| Dining room with storage space for snacks. |  |  |
| Dining room with washbasin for hand hygiene and night light. |  |  |
| Kitchen and pantry. |  |  |
| Laundry. |  |  |
| Space for storing clothes for collective use. |  |  |
| Storage space for cleaning materials. |  |  |
| Undifferentiated warehouse with a minimum area of 10.0 ^m2^ . |  |  |
| Changing room and bathroom for employees, separated by gender. |  |  |
| Dumpster or shelter outside the building for storing waste until collection. |  |  |
| Outdoor uncovered area for coexistence and development of outdoor activities ( solarium with benches, vegetation and others). |  |  |
| Individual bedrooms. |  |  |
| Collective dormitories. |  |  |

| 25 With regard to the areas specified by RDC 283, assess whether your institution offers: | | |
| --- | --- | --- |
|  | YES | NO |
| Bedrooms separated by sex, for a maximum of 4 people, equipped with a bathroom. |  |  |
| Do you provide for a minimum room area according to the number of residents? |  |  |
| 01 person, minimum area of 7.50 m ^2^ , including area for storing the resident's clothes and belongings. |  |  |
| Rooms for 2 to 4 people, with a minimum of 5.50 m2 ^per^ bed, including an area for storing residents' clothes and belongings. |  |  |
| Wake-up lights in bedrooms. |  |  |
| Alarm bell in rooms |  |  |
| Provides a minimum distance of 0.80 m between two beds and 0.50 m between the side of the bed and the parallel wall. |  |  |
| Minimum bathroom area of 3.60 m2 ^,^ with 1 basin, 1 washbasin and 1 shower |  |  |
| Areas for the development of activities aimed at residents with degrees of dependence I and II. |  |  |
| Room for collective activities for a maximum of 15 residents, with a minimum area of 1.0 m2 ^per^ person. |  |  |
| Living room with a minimum area of 1.3 m ^2^ per person |  |  |
| socio-family support activities with a minimum area of 9.0 ^m2^ |  |  |
| Collective bathrooms, separated by sex, with at least one toilet stall that allows the front and side transfer of a person in a wheelchair, in accordance with the specifications of NBR9050/ABNT. |  |  |

| 26 Regarding cleanliness, rate from 1 to 5 how well your institution  [1= very little; 2= little: 3= more or less; 4= very; 5 very much] | | | | | |
| --- | --- | --- | --- | --- | --- |
|  | 1 | two | 3 | 4 | 5 |
| Can you keep environments clean, free of waste and odors incompatible with the activity? |  |  |  |  |  |
| Maintain routines for cleaning and sanitizing items and environments available; |  |  |  |  |  |
| Using products must be registered or notified with Anvisa/MS |  |  |  |  |  |

| **FOOD** | | |
| --- | --- | --- |
| 27 Regarding food: | | |
|  | YES | NO |
| Does your institution guarantee food for the elderly, respecting local cultural aspects, offering at least six meals a day? |  |  |
| **They maintain technical standards and routines available regarding the following procedures:** | YES | NO |
| a) cleaning and decontamination of food; |  |  |
| b) food storage; |  |  |
| c) food preparation with a focus on good handling practices; |  |  |
| d) good practices for vector prevention and control; |  |  |
| e) packaging of waste. |  |  |

| **CLOTHING PROCESSING** | | |
| --- | --- | --- |
| 28 Regarding washing, processing and storage of clothes, the institution: | | |
|  | YES | NO |
| It has a storage space for personal and collective clothing. |  |  |
| It has a technical routine for processing and cleaning clothes. |  |  |
| It allows independent elderly people to carry out all the processing of clothing for personal use. |  |  |
| Promotes the identification of clothes, aiming to maintain individuality and humanization. |  |  |
| Use products registered or notified with Anvisa/MS to wash clothes. |  |  |

| **MONITORING AND EVALUATION OF OPERATION** | | | | | |
| --- | --- | --- | --- | --- | --- |
| 29 Regarding the Health Care Plan, evaluate which items your institution has the MOST EASY to comply  [1= very little; 2= little: 3= more or less; 4= very; 5 very much]: | | | | | |
|  | 1 | two | 3 | 4 | 5 |
| Preparation every two years of the Comprehensive Health Care Plan for residents, in conjunction with the local health manager. |  |  |  |  |  |
| Provide comprehensive health care for the elderly, addressing aspects of promotion, protection and prevention; |  |  |  |  |  |
| Have information about incident and prevalent pathologies in residents. |  |  |  |  |  |
| Annually evaluate the implementation and effectiveness of the actions foreseen in the plan, considering, at a minimum, the criteria of access, resolvability and humanization. |  |  |  |  |  |
| Prove mandatory vaccination of residents as stipulated by the National Immunization Plan of the Ministry of Health. |  |  |  |  |  |
| Respect health surveillance regulations regarding the storage and administration of medicines, and the stock of medicines without a medical prescription is prohibited. |  |  |  |  |  |
| Have written routines and procedures regarding the care of the elderly |  |  |  |  |  |
| In case of medical complications, immediately refer the elderly person to the reference health service provided for in the care plan and inform their family or legal representative. |  |  |  |  |  |
| Provide a removal service designed to transport the elderly; |  |  |  |  |  |

| 30 Assess from 1 to 5 (1 being very little and 5 being very good/excellent) how much your institution CAN MEET the operating conditions set out in article 4 of RDC 283 of 2005.  [1= very little; 2= little: 3= more or less; 4= very; 5 very much]: | | | | | | | | | |
| --- | --- | --- | --- | --- | --- | --- | --- | --- | --- |
|  | | | | 1 | two | 3 | | 4 | 5 |
| Observe the rights and guarantees of the elderly, including respect for freedom of belief and freedom to come and go, as long as there are no restrictions determined in the Health Care Plan. | | | |  |  |  | |  |  |
| Preserve the identity and privacy of the elderly, ensuring an environment of respect and dignity; | | | |  |  |  | |  |  |
| Promote a welcoming environment. | | | |  |  |  | |  |  |
| Promote mixed coexistence between residents of different degrees of dependence. | | | |  |  |  | |  |  |
| Promote the integration of elderly people in activities carried out by the local community. | | | |  |  |  | |  |  |
| Encourage the development of joint activities with people from other generations. | | | |  |  |  | |  |  |
| Encourage and promote family and community participation in care for elderly residents. | | | |  |  |  | |  |  |
| Develop activities that encourage the autonomy of elderly people. | | | |  |  |  | |  |  |
| Promote leisure conditions for the elderly, such as: physical, recreational and cultural activities. | | | |  |  |  | |  |  |
| Develop activities and routines to prevent and curb any type of violence and discrimination against people living there. | | | |  |  |  | |  |  |
| 31 In your opinion, TELL THE CHALLENGES to ensure the proper functioning of LTCs ?  ( ) Availability of financial resources to provide care and general expenses of the institution  ( ) Availability of Human Resources  ( ) Adequacy of the property and facilities of the institution  ( ) Communication with Health Surveillance Supervision  ( ) Communication with bodies defending the rights of elderly people  ( ) Coverage and service of the Unified Health System  ( ) Medical and health monitoring  ( ) Offer of leisure activities  ( ) Guaranteeing the privacy of residents  ( ) Others ____________________________ | | | | | | | | | |
| 32 Of these challenges, CHECK THE THREE that most impact the proper functioning of LTCs ?  ( ) Availability of financial resources to provide care and general expenses of the institution  ( ) Availability of Human Resources  ( ) Adequacy of the property and facilities of the institution  ( ) Communication with Health Surveillance Supervision  ( ) Communication with bodies defending the rights of elderly people  ( ) Coverage and service of the Unified Health System  ( ) Medical and health monitoring  ( ) Offer of leisure activities  ( ) Guaranteeing the privacy of residents  ( ) Others ____________________________ | | | | | | | | | |
| 33 In your opinion, what actions can contribute to the institution achieving the provisions set out in RDC283?  [Check one or more alternatives] | | | | | | | | | |
| ( ) Agreement with the Unified Social Assistance System to increase the institution’s revenue | | | | | | | | | |
| ( ) Training and training of the team of professionals | | | | | | | | | |
| ( ) Partnerships with the third sector | | | | | | | | | |
| ( ) Reforms and adjustments to the physical space | | | | | | | | | |
| ( ) Support from civil society | | | | | | | | | |
| ( ) Strengthening partnerships with the Unified Health System | | | | | | | | | |
| ( ) Approach and better communication with health surveillance bodies | | | | | | | | | |
| ( ) Insertion of professionals with higher education in health | | | | | | | | | |
| ( ) Administrative adjustments | | | | | | | | | |
| ( ) Support from universities and educational institutions; | | | | | | | | | |
| ( ) Partnerships with leisure programs aimed at elderly people; | | | | | | | | | |
| ( ) Support from the elderly’s defense bodies; | | | | | | | | | |
| ( ) Others | | | | | | | | | |
| 34 In the opportunity to contribute to the reformulation of RDC 283, what actions do you propose: | | | | | | | | | |
|  |  | **YES** |  | | | | **NO** | | |
|  | **Emerging** (immediate implementation) | **Urgent** (implementation required, but can wait) | Necessary in the medium and long term. | | | |  | | |
| Compose additional laws that regulate minimum financing for care for institutionalized elderly people by municipalities and states; |  |  |  | | | |  | | |
| co-responsible for actions to strengthen institutions |  |  |  | | | |  | | |
| Reference guidelines for agreeing care for elderly people institutionalized by health units in the region; |  |  |  | | | |  | | |
| Insert into the technical management team at least one professional with knowledge in Gerontology; |  |  |  | | | |  | | |
| Establish supervisory powers for health surveillance, municipal and state councils for the elderly, and the Public Ministry; |  |  |  | | | |  | | |
| Insert monitoring and education actions into the inspection process; |  |  |  | | | |  | | |
| To measure the number of elderly people per caregiver, insert classification criteria based on the complexity of assistance and care; |  |  |  | | | |  | | |
| Others. |  |  |  | | | |  | | |

35. Comments, suggestions and proposals for improving RDC 283 and the inspection process

______________________________________________________________________________
